# 1179. Infectious Diseases Trained Pharmacist: When More Means Less

**DOI:** 10.1093/ofid/ofad500.1019

**Published:** 2023-11-27

**Authors:** Sharon Blum, Meredith Akerman

**Affiliations:** NYU Langone Health - Long Island, Lawrence, New York; NYU Langone - Long Island, mineola, New York

## Abstract

**Background:**

The archived Antimicrobial Stewardship Guidelines from the Infectious Diseases Society of America outline core members of the multidisciplinary team including an infectious diseases trained clinical pharmacist. Evidence exploring impact of infectious diseases (ID) training on a clinical pharmacist's ability to provide effective stewardship are lacking.

**Methods:**

This is a time series data analyzed using control charts examining authorization patterns between ID trained clinical pharmacists and clinical pharmacists without additional ID training covering the antimicrobial stewardship prior authorization and approval requests. U-charts were used to show the proportion of approved antibiotics overall, as well as separately by antibiotic type in subgroups of various sample sizes at 3 time points including: 1. a baseline period of coverage by ID trained clinical pharmacist, 2. a transition to coverage by clinical pharmacists without specific ID training, and 3. a return to the baseline coverage. If any of the points in the chart were outside of ± 3σ limit, then the process was considered out of control at that time point. Analyses were performed using SAS PROC SHEWHART (SAS Institute Inc, Cary, NC).

**Results:**

There was an increase in the rate of approved antibiotics, in the rate of approved Carbapenem, and in the rate of approved Quinolone during the second time period, clinical pharmacists without additional ID training, that lied outside of the ± 3σ limit. The remaining approved antibiotic rates lied within the acceptable ± 3σ limit.

Overall Antibiotic Approvals

**Figure:**
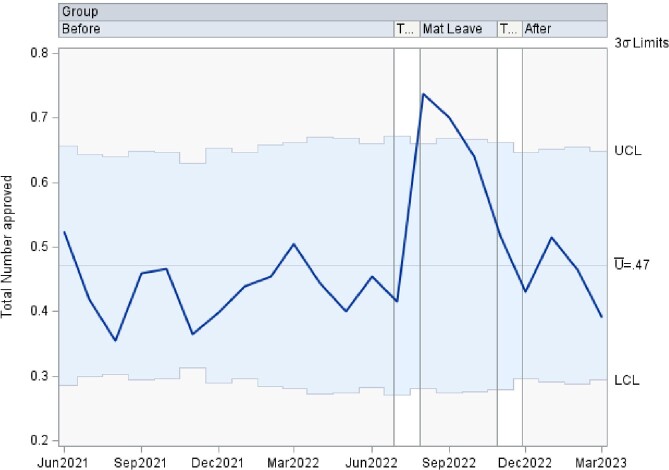

Vancomycin approvals

**Figure:**
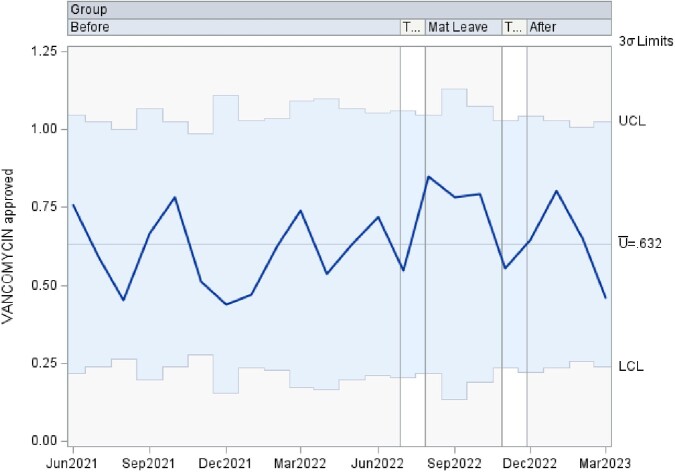

Piperacillin/tazobactam Approvals

**Figure:**
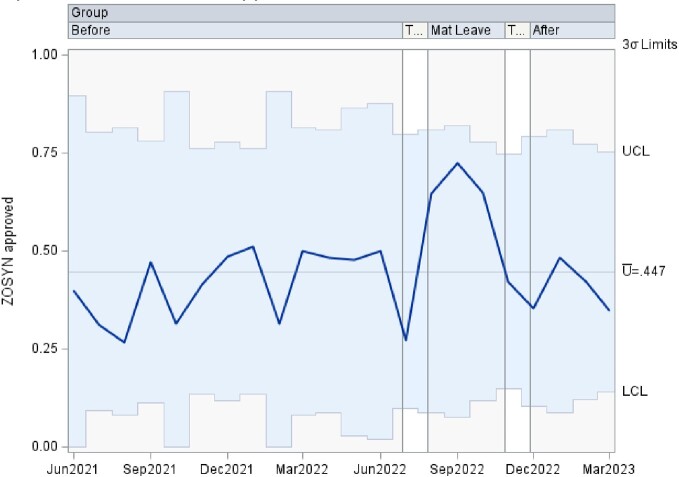

**Conclusion:**

There was a significant difference in antibiotic approvals when Antimicrobial Stewardship coverage was provided by an ID trained clinical pharmacist compared to coverage by clinical pharmacists without additional ID training. ID trained clinical pharmacists were more successful in ability to guide providers to narrower spectrum options or to avoid antibiotic use altogether.

Carbapenem Approvals

**Figure:**
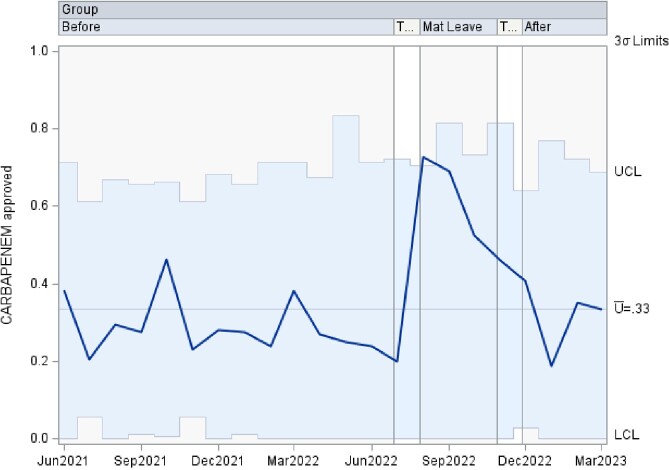

Quinolone Approvals

**Figure:**
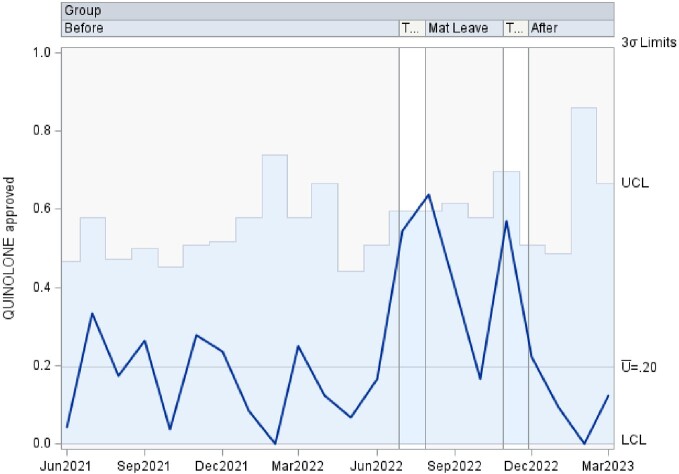

Cefepime Approvals

**Figure:**
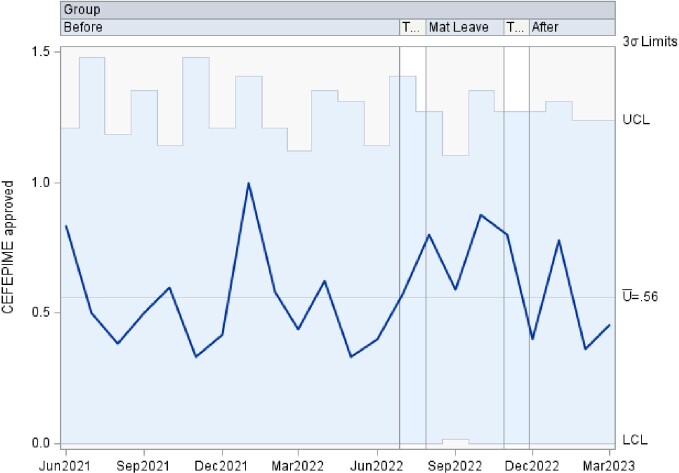

**Disclosures:**

**All Authors**: No reported disclosures

